# Open treatment of dysplasia—other than PAO: does it have to be a PAO?

**DOI:** 10.1093/jhps/hnv028

**Published:** 2015-05-13

**Authors:** Kotaro R. Shibata, Shuichi Matsuda, Marc R. Safran

**Affiliations:** 1. Department of Orthopaedic Surgery, Kyoto University, Kyoto 606-8507, Japan; 2. Department of Orthopaedic Surgery, Stanford School of Medicine, Stanford CA 94063, USA; 3. Chair of Department of Orthopaedic Surgery, Kyoto University, Graduate School of Medicine, Kyoto 606-8507, Japan; 4. Professor of Sports Medicine and Arthroscopy, Department of Orthopaedic Surgery, Stanford School of Medicine, Stanford, CA 94063, USA

## Abstract

Hip dysplasia is a developmental disorder that results in anatomic abnormalities in which the acetabular coverage is insufficient. In the absence of severe degenerative changes, younger active patients with these symptomatic structural abnormalities are increasingly managed with joint-preserving operations. Historically there have been numerous reconstructive pelvic osteotomies. In recent years, the Bernese periacetabular osteotomy (PAO) has become the preferred osteotomy by many surgeons. Even so, as our understanding of the hip advances and new diagnostic and treatment techniques are developed, we sought to put a focus on the long-term results of augmental osteotomies and pelvic osteotomies other than the PAO, to see if any of these surgeries still have a place in the current algorithm of treatment for the dysplastic hip. As the longevity of the treatment is the focal point for joint preservation surgeries for the dysplastic hip, these authors have searched databases for articles in the English literature that reported results of long-term follow-up with a minimum of 11-year survivorship after surgical treatment of developmental dysplasia of the hip. Reconstruction osteotomies for the dysplastic hip are intended to restore normal hip anatomy and biomechanics, improve symptoms and prevent degenerative changes, in this manuscript each procedure is independently assessed on the ability to achieve these important characteristics.

## INTRODUCTION

Hip dysplasia is a developmental disorder that results in anatomic abnormalities in which the acetabular coverage is insufficient, predisposing the hip joint to have increased contact pressure on the cartilage as well as instability which leads to damage to the soft tissue structures surrounding the joint, eventually leading to coxarthrosis. In the absence of severe degenerative changes, younger active patients with these symptomatic structural abnormalities are increasingly managed with joint-preserving operations with the goal of improving function and possibly preventing the development of severe degenerative changes. For this purpose, many variations of augmental osteoplasties and pelvic osteotomy surgeries have been proposed.

Historically in adolescents with a closed triradiate cartilage and in young adults, there have been numerous reconstructive pelvic osteotomies, with each surgery aiming to address the problems of the earlier generation procedures, in an attempt to improve on the results (Table I). In recent years, because of its ability to correct the deformity, its inherent stability and blood supply, the Bernese periacetabular osteotomy (PAO) [1] has become the preferred osteotomy by many surgeons in North America and Western Europe. Even so, as our understanding of the hip advances and new diagnostic and treatment techniques are developed, we sought to put a focus on the long-term results of augmental osteotomies and pelvic osteotomies other than the PAO, to see if any of these surgeries still has a place in the current algorithm of treatment for the dysplastic hip. As the longevity of the treatment is the focal point for joint preservation surgeries for the dysplastic hip, these authors have searched databases for articles in the English literature that reported results of long-term follow-up with a minimum of 11-year survivorship after surgical treatment of developmental dysplasia of the hip (DDH).

**Table I.** Evolution of Pelvic osteotomies

Type	Surgeon	Year
Shelf	Köng	1891
	Albee	1915
	Spitzy	1923
Acetabuloplasty	Penberton	1965
	Dega	1974
Chiari	Chiari	1953
Single	Salter	1961
Double	Sutherland and Greenfield	1977
Triple	LeCour	1965
	Hopf	1966
	Steel	1973
Triple Juxta-artilular	Tönnis	1977
	Carlioz	1982
Spherical	Wagner	1976
Dial	Eppright	1975
Rotational	Tagawa and Ninomiya	1982
Bernese periacetbular	Ganz	1983

Reconstruction osteotomies for the dysplastic hip are intended to restore normal hip anatomy and biomechanics, improve symptoms and prevent degenerative changes. Theoretically, the ideal osteotomy should be as non-invasive as possible, technically reproducible, have sufficient graft mobility, have the ability to increase the area of weight bearing hyaline cartilage, medialize the center of rotation, have reliable blood supply for the graft, be a stable osteotomy, and have a broad range of indications. In this manuscript, each procedure is independently assessed on the ability to achieve these important characteristics.

### Salter innominate osteotomy

**Fig. 1.**
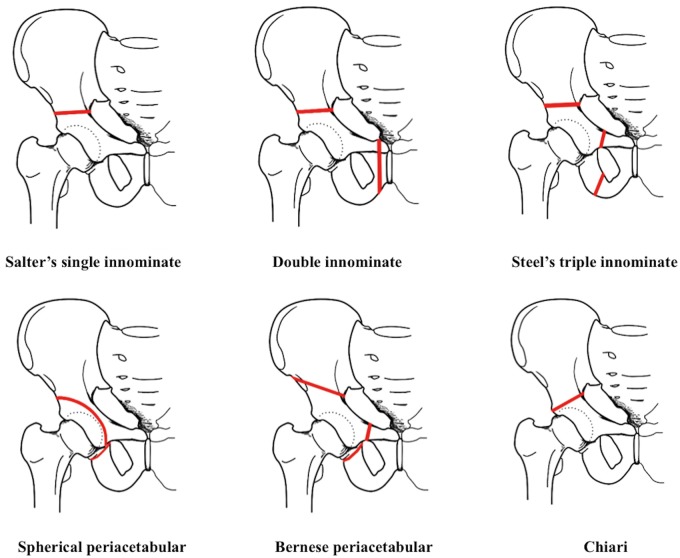
Pelvic osteotomies for hip dysplasia. Red lines indicate site of osteotomy.

Salter introduced the single innominate osteotomy in 1961 [2]. Salter and others [3, 4] observed that the acetabulum in the congenitally dislocated or subluxated hip-faced abnormally anterolaterally. For purpose of correcting this a single innominate osteotomy is made from the sciatic notch to the anterior inferior spine, using the symphysis pubis as a pivot the innominate bone containing the entire acetabulum is shifted forwards, downwards and outwards so that the osteotomy site is open anterolaterally, and the femoral head coverage is increase and dislocation or subluxation is reduced [2] (Fig. 1). Salter noted that the osteotomy is inherently stable, and that early weight bearing possible, and in fact, a vital stimulus for remodeling and further osseous development of the components of the hip. For this he emphasized the importance of conducting the surgery while there is still sufficient remodeling potential remaining [2].

Because of its simplicity and its wide spread use in the 1970’s, the Salter innominate osteotomy was also utilized in adult patients. But because the osteotomy relies on the symphysis pubis as a pivot, the obtainable degree of correction in an adult patient was limited. The center of rotation of the hip is also moved laterally, which increases the stresses over the hip joint causing concern in the adult patient. The surgery has since lost its place in the treatment of the skeletally mature patient.

The long-term results of the Salter single innominate osteotomy have exclusively been reported in the young child, with many series reporting excellent results. Böhm *et al*. [5] and Thomas *et al*. [6] have presented 26–35 and 40–48 year follow-ups of patients who were treated at a mean age of 4.1 years old and 2.8 years old, respectively. The survival rate with Total Hip Arthroplasty (THA) as an end point was 79% at 31 years for Böhm and 99% at 30 years, 86% at 40 years and 54% at 45 years for Thomas’ study group (Table II). The procedure has since become the gold standard for treatment of DDH in children, preferably under the age of 6 [2].

**Table II.** Survivorship of the hip after Salter’s single innominate osteotomy

Authors	Year	Technique	No. of hips	Mean age (year)	Duration of follow-up (year)	Survival rate (%) (THA as endpoint)	Survival rate (%) (poor results as an endpoint)	Follow-up rate (%)
Böhm and Brzuske [5]	2002	Salter	74	4.1 (1.3–8.8)	31 (26–35)	90	79	100
Thomas *et al*. [6]	2007	Salter	101	2.8 (1.5–4.7)	43 (40–48)	70	99 at 30 years, 86 at 40 years, 54 at 45 years	79

#### In assessing the Salter innominate osteotomy

Many of the ideal theoretical goals are achieved. It is a fairly non-invasive and reproducible surgery. There is increase of the weight bearing area of Hyaline cartilage. The movability of the graft is constrained and medialization cannot be obtained. The osteotomy is stable and the blood supply to the graft is good, and there is limited narrowing of the pelvic ring. The level of dysplasia that can be treated with this technique is limited, however, and can only be use effectively in young children (Table VIII).

### Acetabuloplasty

The Acetabuloplasty operations are operations exclusively done in children for the treatment of DDH. These operations should be noted for their historic importance in the evolution of pelvic osteotomy, but because they are surgical techniques that cannot be done in the skeletally mature patient, they are not considered as an alternative to the PAO. As such, these operations will only briefly be reviewed. Acetabuloplasty procedures are operations designed to change the slope of the roof of the acetabulum by making an incomplete osteotomy above the joint and hinging down the graft with the acetabular roof and insertion of a bone graft to keep the osteotomy site open [7, 8]. Pemberton [8] introduced his acetabuloplasty in 1965, which he called osteotomy of the ilium with rotation of the acetabular roof. The osteotomy extends through the inner cortex of the ilium up to the posterior arm of the triradiate cartilage, and the iliac portion of the acetabulum is hinged forward on the triradiate cartilage, allowing for a change in both the volume and orientation of the acetabulum. As it is not possible to change the slope of only the iliac part of the articular surface, ultimately the internal congruity of the joint is altered and degenerative changes in the joint may be a concern [9]. Risk of avascular necrosis and premature closure of the triradiate cartilage are also concerns with these techniques [8, 10]. Two long-term reports both showed excellent to good results in the young child [11, 12].

### Double innominate osteotomy

The double innominate osteotomy was introduced by Sutherland and Greenfield [13]. This surgery added to Salter’s technique an osteotomy of the pubis, medial to the obturator foramen, making it possible to rotate the acetabular fragment even in older patients. Excising extra bone from the pubis also permits medial displacement of the fragment, achieving a biomechanical advantage (Fig. 1). But because the acetabular fragment is large, when major correction is performed, rotation of the fragment leaves a considerable defect at the osteotomy site. Mobility of the fragment is limited by muscular and sacroiliac ligament connections, limiting fragment movement to a lateral and anterior rotation. The procedure did not achieve wide spread usage. We know of only one mid-term follow-up of 21 patients for a mean of 4.9 years, from Dr Sutherlands group. The mean age of the patients was 13.1 (range 6–27) years old. At follow-up, a statistically significant improvement in pain level, functional status, acetabular index, center-edge angle and relative lateral displacement was observed [14].

#### Assessing the double pelvic osteotomy

Compared with the Salter osteotomy it is a more invasive surgery. There is increase of the weight bearing area of Hyaline cartilage. The movability of the graft is still constrained and optimal medialization can be difficult. The osteotomy is stable the blood supply to the graft is stable, and the narrowing of the pelvic ring is limited. The level of dysplasia that can be treated with this technique is limited (Table VIII).

### Triple innominate osteotomy

Triple innominate osteotomies were described to overcome the drawbacks of the Salter innominate osteotomy, especially addressing the restricted movement of the fragment and the lateralization of the hip joint. The basis of this operation was to make an osteotomy of the pubis and ischium, to allow for more freedom to move the fragment. The operations described by LeCoeur [15], Hopf [16] and Steel [17] are all variations on the theme.

LeCoeur was the first to publish a technique for triple osteotomy of the innominate bone. The method divides the pubis and the ischium close to the symphysis pubis crossing the medial lesion of the obturator foramen. One year later Hopf published a technique that allowed all three osteotomies to be performed through a single Smith-Petersen approach. For more cephalad dislocations, Hopf recommended the osteotomy go through the floor of the true acetabulum, osteotomizing both the ilium and the ischium together. This was in concept a triple osteotomy done by a double osteotomy. But as the osteotomy puts the vascularization of the acetabulum at risk, there was concern of osteonecrosis of the graft [18]. Steel’s osteotomy is more distal from the acetabulum crossing the obturator and it is performed through three separate incisions (Fig. 1). Though there was improvement in the movement of the graft, in a large correction these osteotomies still were limited by the size of their fragments, the muscular attachments and the ligamentous connections to the sacrum, particularly the Sacrospinous ligament, also significant corrections resulted in notable asymmetry of the pelvis.

Tönnis *et al*. [19] and Carlioz *et al*. [20] addressed these problems by describing a juxta-articular triple innominate osteotomy. By conducting the ischial osteotomy proximal to the ischial spine it made it possible to avoid the connection to both the Sacrotuberous ligament and the Sacrospinous ligament, considerably increasing the three dimensional mobility of the graft. Although this avoided the restricting ligaments, it still had its drawbacks as the osteotomies went through the posterior column creating a pelvic discontinuity, making the acetabular fragment and the pelvic ring unstable. Rigid fixation and long-term postoperative immobilization usually in a cast, was necessary.

Four long-term reports have reviewed the results of follow-ups that are more than a mean of 11 years [21–24] (Table III). Dungl *et al*. [22] reported the results of a modified Steel’s triple osteotomy technique in 351 hips (329 cases). There were 146 hip joints in 128 patients (76%) with excellent results in the group of congruent hips without preoperative arthrosis. In 182 hips in 178 patients with hip joints with some deformity, limited ROM and decentration, 40% were excellent, 32% good, 23% fair and 5% unsatisfactory results were achieved. They noted a 5.4% rate of nonunion at the osteotomy site.

**Table III.** Survivorship of the hip after Triple innominate osteotomy

Authors	Year	Technique	No. of hips	Mean age (year)	Duration of follow-up (year)	Survival rate (%) (THA as endpoint)	Survival rate (%) (poor results as an endpoint)	Follow-up rate (%)
Guille *et al*. [21]	1992	Triple	11	14 (11–16)	12 (10–16)	91	91	100
Dungl *et al*. [22]	2007	Triple (Steel)	351	16.5 (9–41)	12.5 (4–25)	95	94	91
Janssen *et al*. [23]	2009	Triple (Tönnis)	35	38.6 (23.9–57)	11.5 (11–12.2)	85.3	78	100
van Stralen *et al*. [24]	2013	Triple (Tönnis)	48	28 (14–48)	25 (23–29)	67	83 at 10 years, 81 at 15 years, 65 at 25 years	96

Jannsen *et al*. [23] assessed the long-term results of Tönnis’s triple osteotomy in patients with advanced osteoarthritis of the hip, 35 patients with a mean age of 38.6 years (range 23.9–57 years) and follow-up of 11.5 years (range 11–12.2 years). Conversion to a total hip replacement as end point, revealed a survival rate of 85.3% (95% confidence interval 74.1–98.1%) at the time of the last follow-up examination. van Stralen *et al*. [24] assessed the long-term results of Tönnis’s triple osteotomy in 51 pelvic osteotomies in 43 patients (38 females and five males; mean age, 28 years; range, 14–48 years). The minimum follow-up was 23 years (mean, 25 years; range, 23–29 years). The survival rate was 94% at 10 years, 88% at 15 years and at 20 years 76% using THA as an end point.

#### Assessing Steel’s triple pelvic osteotomy

It is an invasive surgery. There is increase of the weight bearing area of Hyaline cartilage. The movability of the graft is much improved but still constrained and obtaining optimal coverage and medialization can be difficult. The osteotomy is unstable needing postoperative immobilization. Besides the Hopf technique, the blood supply to the graft is stable, considerable deformity of the pelvic ring is expected in cases with large corrections. The level of dysplasia that can be treated can be limited (Table VIII).

#### Assessing the juxa-articular triple pelvic osteotomy

It is an invasive surgery. Weight bearing area of Hyaline cartilage is increased. The movability of the graft is much improved. The osteotomy is unstable needing postoperative stabilization. The blood supply to the graft is stable. Considerable deformity of the pelvic ring is expected in cases with large corrections. The level of dysplasia that can be treated is good (Table VIII).

### Spherical periacetabular osteotomies

Spherical periacetabular osteotomies include the Spherical osteotomy described by Wagner [25], the Dial osteotomy described by Eppright [26] and the Rotational acetabular osteotomy (RAO) described by Ninomiya and Tagawa [27]. There are slight differences in the surgical technique between the surgeons but in general, using curved osteotomes, taking care not to extend into the joint, and with the inner cortical table and posterior column kept intact, a spherical or circumferential osteotomy close and around the acetabulum is made. Once completed, without losing stability of the pelvic rim, a free acetabular fragment is released allowing for an individualized correction of the joint position. This was advantageous compared with the other innominate osteotomies, as it has no tethering ligaments or muscle attachments allowing for large corrections while leaving the posterior column intact for stability of the pelvis (Fig. 1).

Even though the surgery is highly advantageous when considering the ability to reposition the acetabulum, there are a few inherent drawbacks of the surgery that must be considered. Because of the proximity of the osteotomy to the joint medially and distally, part of the osteotomy is actually intra-articular, which makes the vascular supply to the acetabular fragment reliant on the vascularity through the undamaged hip joint capsule, making it difficult to explore or treat pathology inside the joint [28–32]. Furthermore, the proximity of the osteotomy to the joint makes this osteotomy a technically demanding procedure, with considerable risk of unintended penetration in to the joint by the curved osteotomy. Also because the quadrilateral plate largely remains intact, medialization of the fragment is limited [31, 33, 34].

Though there have only been a few long-term reports on the spherical and dial osteotomies [33, 35]. There have been many reports on the RAO exclusively from Japanese surgeons [32, 36–41], as historically the Japanese have been known to have a high rate of hip dysplasia, and the treatment and prevention of DDH has rendered much concern over the years. Ninomiya and Tagawa [27] first reported on the RAO technique in the Japanese literature in 1982 and then in the English literature in 1984. Their excellent results have led many Japanese surgeons to incorporate their technique and surgery, making it a main stay in Japanese orthopedics.

We identified six studies [32, 36, 38, 42–44] reporting on the long-term results of the RAO (Table IV). The mean postoperative follow-up period of these reports was 11–15 years, the age at operation ranged from 11 to 59 years old and the mean age ranging from 28 to 47 years old. The survival rate with THA as an end point ranged from 87 to 100% and with clinical failure as an end point 68 to 90%. The rate of major complications such as bone graft necrosis, intra-articular damage, postoperative loss of coverage, pulmonary embolism ranged from none to 9.6%, depending on the surgeon.

**Table IV.** Survivorship of the hip after Spherical periacetabular osteotomies

Authors	Year	Technique	No. of hips	Mean age (year)	Duration of follow-up (year)	Survival rate (%) (THA as endpoint)	Survival rate (%) (poor results as an endpoint)	Follow-up rate (%)
Schramm *et al*. [33]	2003	Spherical, Wagner	22	24.4	23.9 (22–29.3)	76	86 at 20 years, 76 at 24, 65 at 25 years	88
Miller *et al*. [34]	2005	Dial	44	18.9 (8–31)	12.6 (5.6–20.2)	87	73	N/A
Nakamura *et al*. [36]	1998	RAO	145	28 (11–52)	13 (10–23)	95	68	63
Nozawa *et al*. [46]	2002	RAO	50	31.8 (13–53)	11.4 (10–14.5)	98	80	90
Yasunaga *et al*. [40]	2004	RAO	61	35 (13–58)	10.5 (8–14.5)	100	90	95
Okano *et al*. [43]	2008	RAO	49	33 (13–54)	13 (10–17)	100	81	82
Hasegawa *et al*. [38]	2014	ERAO	130	27 (15–59)	19.7(15-23)	87	78	98
Ito *et al*. [44]	2011	RAO	117	Young 27 (12–39)	11 (5–20)	97	87	95
			41	Old 47 (40–56)	11 (5–19)	93	73	95

The mean preoperative center edge (CE) angle ranged from −1.7° to 3° and postoperatively 35° to 43°. The preoperative severity of the CE angle did not significantly influence any of the results. Negative factors influencing the results of the RAO were a higher age, the degree of osteoarthritis and not being a congruent joint at the time of surgery. The congruency of the joint was confirmed by the ability to obtain good congruity in the hip abduction position.

There have also been many reports from Japan [39, 41, 44–46] showing that even in older patients and patients with preexisting OA, that the RAO can be beneficial in the Japanese population.

Overall, the long-term results of the RAO in these series were excellent, and the complication rates, particularly of fragment vascularization related problems, were low. However, there have been other reports of a high rate of acetabular graft necrosis and chondrolysis after RAO [30]. The concerns of osteonecrosis of the acetabulum seem to be low in the hands of the experienced surgeon. Surgical technique must be taken into particular consideration when conducting this operation.

All of the excellent long-term results of RAO have come from Japan; there is a possibility that the small stature and lighter weight of the Japanese and also the strong negativity toward prostheses at a young age in the Japanese culture may have influenced some of the results.

#### Assessing the spherical periacetabular osteotomies

It is an invasive surgery. Weight bearing area of Hyaline cartilage is increased. The movability of the graft is good. Medialization of the join is restricted. The osteotomy is stable. The blood supply to the graft is a concern. The pelvic ring is maintained. The level of dysplasia that can be treated is good (Table VIII).

### Shelf operation

**Fig. 2.**
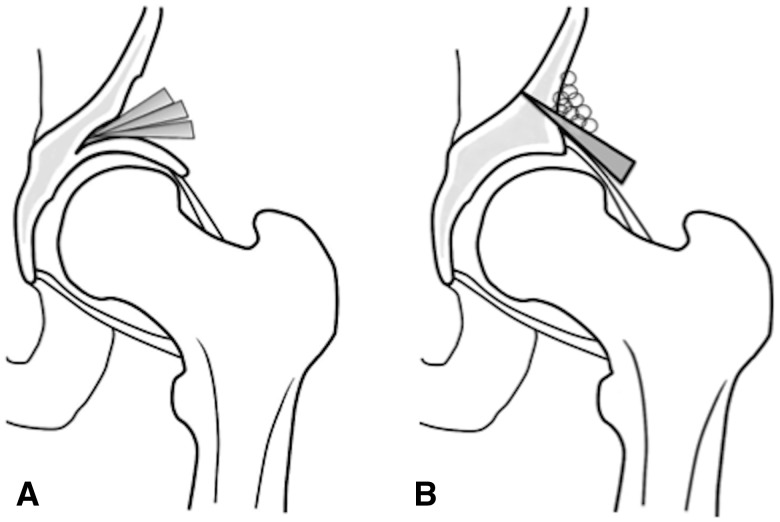
Shelf operation.

The Shelf operations were designed to increase coverage of the femoral head by constructing an extra-articular bony buttress extension of the slope of the acetabulum, and was first described by Köng [47]*.* Since then many surgeons have advocated variations of the surgery. For the skeletally immature patients a technique more similar to an acetabuloplasty has been used [48–53] (Fig. 2A). In the skeletally mature patient, the Spitzy and Bosworth type techniques have been extensively used [54–60] (Fig. 2B). In short, commonly utilizing the Smith-Peterson approach the external aspect of the anterior ilium and capsule of the hip joint is exposed. A buttress autograft is placed close to the joint margin and directed downward and anterolaterally on top of the joint capsule. The bone graft is then stabilized by wedging it into a slot made above the acetabular margin or by screw or pin fixation, and various forms of bone grafting techniques are applied over the Shelf for reinforcement and bone union. The Shelf reinforces the fibrous capsule of the joint that prevents lateral and upward subluxation of the femoral head. Chiari [61] hypothesized, and Hiranuma *et al*. [62] has reported in rabbits, that the interposed capsular tissue undergoes metaplastic change to fibrocartilaginous tissue. It is believed that the same metaplastic change occurs in the interposed capsule of the Shelf and plays an important role in the formation of a new weight bearing area [63]. Postoperative management is time consuming, as recovery from the damage to the abductor muscles and bony fixation of the graft is needed before full weight bearing is possible. Even then, the long-term affect of the damage to the abductors may reside.

The Shelf procedure is the oldest surgery advocated for the dysplastic hip and initially the most utilized procedure for this entity [64]. As the true indications for the surgery were not known and undoubtedly owing to the relatively uncomplicated nature of the operation, it was utilized for various types of dysplasia of the hip from the infant with DDH to the older patient with osteoarthritic dysplasia. Because of this many of the long-term reports of the Shelf procedure have included a very heterogeneous group of patients, as the results have been mixed, it has consequently made it difficult to identify the relevance and the true indications of this technique.

There have been several reports of poor long-term results after the Shelf operation [65–67] (Table V). White *et al*. [65] reported that poor results were seen in 57% of the patients at 22 years follow-up. Miguad *et al*. [67] reported on a 58% survival rate at 15 years follow-up with THA as an end point. Fawazy *et al*. [66] reported a survival rate of 46% at a mean follow-up of 11 years with THA as an end point. But a detailed look at the patients in each cohort shows that, all three reports are follow-ups of very difficult cohorts of patients. The patients in White *et al*.’s series were all congenital subluxations that required intra operative reduction and traction treatment after the Shelf procedure. Migaud *et al*.’s cohort was a group of patients that all had more than a Grade 2 degree of osteoarthritis, and 57% had more than Grade 3–4 osteoarthritis and 50% of the cohort had subluxation of the femoral head. Fawazy *et al*. reported on a group, which included 42% with Grade 4 osteoarthritis.

**Table V.** Survivorship of the hip after Shelf osteotomy

Authors	Year	Technique	No. of hips	Mean age (year)	Duration of follow-up (year)	Survival rate (%) (THA as endpoint)	Survival rate (%) (poor results as an endpoint)	Follow-up rate (%)
White *et al*. [65]	1980	Shelf	29	7	22 (10–31)	43	57 at 25 years	N/A
Fawzy *et al*. [66]	2005	Shelf	76	33 (17–60)	11 (6–14)	46	86 at 5 years, 46 at 10 years	100
Miguad *et al*. [67]	2004	Shelf	56	32 (17–56)	15 (15–30)	58	37 at 20 years,	93
Bickel and Breivis [68]	1975	Shelf	141	N/A	12.8 (1–32)	94	78	84
Saito *et al*. [70]	1986	Shelf	27	25 (11–55)	12 (5–19)	92	93	77
Summers *et al*. [71]	1988	Shelf	35	14 (3–41)	16 (8–30)	92	67	77
Love *et al*. [69]	1980	Shelf	45	12 (6–22)	11 (2–20)	88	84	80
Nishimatsu *et al*. [73]	2002	Shelf	106	25	23.8 (15–41)	89	70	64
Hirose *et al*. [63]	2011	Shelf	28	34 (17–54)	25 (20–32)	80	78 at 10 years, 53 at 15 years	49
Bartonicek *et al*. [72]	2012	Shelf	25	31 (16–52)	15 (10–23)	80	80	100

The indications for the Shelf operation recently have been drawn from the unsatisfactory results from these and other past studies. It has been learned that the young patient under the age of 10 with congenital dislocation, and hips with subluxation and significant amount of osteoarthritis are susceptible to unsatisfactory results with this technique. Bickel and Brevis [68] reported that although some children <10 years old may have benefited, this age group had the highest percentage of failures of the Shelf operation, with Shelf resorption being a major cause. In fact, when the Shelf procedure had been carried out on patients over the age of 10 years old, no resorption of the graft was observed [59, 68, 69].

Studies which reported on a more homogenous group of patients, where patients’ mean age were over 12 years old with a minimal amount of osteoarthritic change, the results have been good [63, 69–73] (Table V). It should also be noted that the preoperation level of dysplasia in each cohort was not an indication for exclusion of the surgery. Nishimatsu *et al*. [73], Hirose *et al*. [63] and Bartoníček *et al*. [72], each reported on a cohort of patients were the mean age of the patients were 25, 34 and 31 years old and the mean follow was notably long at a mean of 23.8, 25 and 15 years, respectively. Nishimatsu *et al*. reported that 87% of patients whom had pre-stage and initial stage of osteoarthritis (according to the Japanese Orthopaedic Association score) preoperatively had a good clinical result at a mean of 23.8 years follow-up. The mean preoperative and postoperative CE angles were −0.089° (SD 14.8) and 40.3°, respectively. They also determined that the Shelf height was significantly lower in the group with good results compared with the group with poor results. Hirose *et al*. followed 28 patients with a mean preoperative and postoperative CE angles of −4° (−27° to 20°) and 38° (13° to 60°), respectively, and observed a survival rate of 80% at 25 years, but their follow-up rate was low at 49%. Bartoníček *et al*. reported in a subgroup of 20 patients with a mean preoperative and postoperative CE angles of 16° (−15° to 20°) and 32° (25° to 45°), respectively that had a spherical, concentric hip without osteoarthritic change before operation had a survival rate of 94% at a mean follow-up of 14 years.

Judging from the literature, good long-term results can be expected with the Shelf operation and best results may be expected in the young adult with a dysplastic spherical centered hip without osteoarthritic changes. The Shelf operation still has to be considered as a treatment option for the dysplastic hip as long as the indications are thoroughly considered.

#### Assessing the Shelf operation

It is considered to be one of the least invasive surgeries, though the damage to the abductors is a concern. The weight bearing area of Hyaline cartilage cannot be increased. The acetabular joint is not moved. Medialization of the joint cannot be done. The Shelf is stable. The osteonecrosis of the acetabulum is not a concern but resorption of the graft can occur. The pelvic ring is maintained. The level of dysplasia that can be treated is controversial (Table VIII).

### Chiari osteotomy

In the early 1950’s Dr Chiari realized that patients with DDH that had subluxation and/or advanced stage osteoarthritis suffered unsatisfactory results with the Shelf procedures. He indicated that any persistent subluxation after a Shelf operation caused an insufficiency of the gluteal muscles because of the lateralization of the femoral head. To exclusively treat these difficult cases he introduced his medial displacement iliac osteotomy. The osteotomy was introduced in Vienna in 1953, but was only reported in the English literature in 1974 [61]. The basic concept underlying the procedure consists in constructing a congruent shelf above the intact hip joint without bone grafting, and in optimally correcting the pathologic position of the femoral head. This concept is realized by an osteotomy just above the intact joint along a curved line through the iliac isthmus close to the superior insertion of the capsule. The osteotomy extends from under the inferior spine anteriorly to the sciatic notch posteriorly. Lateralization of the hip joint is corrected by pushing the acetabulum medially. The displacement can be done at any age by hinge-type movement at the symphysis (Fig. 1). With this technique abduction is increased, and, although head coverage by the true acetabulum is decreased, coverage is augmented by part of the femoral head articulating with the underlying capsule tissue. The medial displacement osteotomy of the pelvis distributes the load over a more extensive surface area in a larger socket, while at the same time reducing the load by changing the leverage [61].

The surgical advantages are the abnormally lateral position of the hip is overcome, and load upon the hip is reduced through medial displacement. In addition remodeling of the new acetabulum in the postoperative years adds substantially to the area of support for the femoral head as the surface formed at osteotomy becomes more closely applied to the capsule. The operation can accommodate a significant degree of acetabular migration [74, 75].

The Chiari osteotomy historically has been used as a salvage surgery. The indications from the literature are vast; the age indication is from children over the age of 4 with no upper age limit, congenital subluxation of the hip, Coxa magna after Perthes’ and dysplastic hip with or without osteoarthritis.

There have been many series reporting the long-term results of the Chiari osteotomy, and all have shown good results [67, 76–81]. It should be emphasized again, that in all the studies, the indication for the surgery varied widely. Looking exclusively at reports with a mean follow-up of more than 13 years and follow-up rate of better than 69%, the survival rate with THA as an end point ranged from 68 to 97%. The largest cohort of patients was reported by Windhager *et al*. [77]. They reviewed 236 Chiari Osteotomies performed between 1953 and 1967. Twenty-one hips needed reoperation after an average of 15.4 years; the other 215 hips had been followed up for 20–34 years (mean 24.8 years). Clinical results were excellent or good in 51.4%, fair in 29.8% and poor in 18.3%. The results were worse in patients with increased age and signs of osteoarthritis preoperatively. A consistent loss of hip joint range of motion was observed, which was 15°, 13°, 13°, in flexion, internal rotation and external rotation, respectively. Additionally, the Trendelenburg sign rarely showed signs of improvement when the operation was done in patients over the age of seven [77] (Table VI).

**Table VI.** Survivorship of the hip after Chiari osteotomy

Authors	Year	Technique	No. of hips	Mean age (year)	Duration of follow-up (year)	Survival rate (%) (THA as endpoint)	Survival rate (%) (poor results as an endpoint)	Follow-up rate (%)
Calvert *et al*. [76]	1987	Chiari	52	19.8 (3–41)	14 (10–19)	94	65	72
Windgager *et al*. [78]	1991	Chiari	236	14.1 (2.6–51.3)	25 (20–34)	91	82	60
Lack *et al*. [77]	1991	Chiari	100	38 (30–59)	16 (10–21)	80	64	70
Ohashi *et al*. [80]	2000	Chiari	103	18.2 (6–48)	17 (4–37)	94	84 at 10 years, 68 at 20 years	81
Miguad *et al*. [67]	2004	Chiari	89	33 (17–56)	18 (6–25)	68	78 at 13 years	93
Yanagimoto *et al*. [79]	2005	Chiari	74	32 (6–64)	13 (10–20)	97	90	69
Kotz *et al*. [81]	2009	Chiari	80	23 (2–50)	32 (27–48)	60	N/A	15

Even though the Chiari osteotomy is simple, safe and a good indication for treatment of pain, its current use is limited. Limitations of the Chiari osteotomy include the fact that it does not restore the joint anatomy and there is a consistent loss of hip joint range of motion. Additionally, the Chiari osteotomy affects the hip abductors, which results in a high rate of residual hip abductor weakness. The Chiari osteotomy is now used mainly where other pelvic osteotomies or acetabuloplasties are ruled out, for neglected congenital dislocation or the hip with or without secondary osteoarthritis as a salvage procedure [78].

#### Assessing the Chiari osteotomy

It is considered to be one of the more non-invasive surgeries. The weight bearing area of Hyaline cartilage cannot be increased. The acetabular joint is moved medially and the medialization of the joint is one of the advantages of the surgery. The osteotomy is stable. The blood supply of the osteotomy is not a concern. The pelvic ring is deformed and of concern if the correction is large. The level of dysplasia that can be treated is broad (Table VIII).

### Periacetabular osteotomy

The Bernese PAO was introduced by Ganz *et al*. [1]. It was developed to theoretically improve on many of the shortcomings of the previously described surgeries by simultaneously improving on features such as the mobility of the graft, which allows for large corrections in all directions including medialization, preservation of the stability of the pelvis by keeping the posterior column intact, assuring good vascularization of the bone graft via preservation of the inferior gluteal artery, which also makes it possible to safely conduct a capsulotomy of the hip joint without risk of devascularization of the bone graft and preserving the true shape of the pelvis ring. The surgery can also be conducted with out violating the abductor mechanism [1, 18]. Even though it is a technically demanding and an invasive surgery, the many advantages of the PAO has resulted in it becoming the preferred procedure by many surgeons for treatment of the dysplastic hip in the skeletally mature patient.

Describing the surgery in short, using an anterior approach and performing most of the osteotomies from the inner aspect of the pelvis, a partial osteotomy of the ischium, a complete osteotomy of the pubis and preserving the continuity of the posterior column, a biplanar osteotomy of the ilium is done. The freed acetabular graft is displaced medially and rotated anterior laterally and fixed with screws. Then the joint capsule can be opened and inspected allowing for treatment of pathology inside the joint [1] (Fig. 1). Although the majority of surgeons performing PAO has traditionally performed capsulotomies to assess the intra-articular milieu, there has been recent enthusiasm for performing concomitant arthroscopy with the PAO, reducing the need for capsulotomies [82, 83]. In fact, it seems there is a trend for performing a minimally-invasive PAO combined with arthroscopic treatment of however, we know of no long-term studies of this technique that have been published in the English literature.

The preliminary surgical results with this technique were first reported by Ganz *et al*. [1] in a mixed group of 75 hips in 63 patients with and without secondary degenerative changes. The average age at surgery was 29 years old. There was marked improvement of pain and excellent femoral head coverage in both the frontal and sagittal planes. There actually have not been many long-term reports that consist of patient follow-ups of a minimum of 11 years besides the reports from the Ganz group. Siebenrock *et al*. [84] and Steppacher *et al*. [85] have up dated the report on the initial 75 hip in 63 patients reported by Ganz *et al*. at a mean follow-up of 11.3 and 20.4 years, respectively. These authors noted that excellent correction was maintained, and the hip joint was preserved in 87.6% at 10 years, 77.3% at 15 years and 60.5% at 20 years. Several factors were associated with an unfavorable outcome, including older age of the patient at the time of the surgery, moderate to severe preoperative coxarthrosis, an associated labral lesion, less anterior coverage correction and a suboptimal postoperative acetabular index [86]. The only other long-term report on the PAO that is over a mean of 11 years is by Kralj *et al*. [87]. They reported on 26 hips at a mean follow-up of 12 years, the survival rate with THA as end point was 85%, but the follow-up rate was 64% (Table VII).

**Table VII.** Survivorship of the hip after Bernese PAO

Authors	Year	Technique	No. of hips	Mean age (year)	Duration of follow-up (year)	Survival rate (%) (THA as endpoint)	Survival rate (%) (poor results as an endpoint)	Follow-up rate (%)
Kralj *et al*. [87]	2005	Bernese	26	34 (17–50)	12 (7–15)	85	54	64
Siebenrock *et al*. [84]	1999	Bernese	75	29.3 (13–56)	11.3 (10–14)	82	73 were good or excellent	95
Steppacher *et al*. [85]	2008	Bernese	75	29.3 (13–56)	20.4 (19–23)	60	57 at 20 years	93

**Table VIII.** Assessing the characteristics of the osteotomies

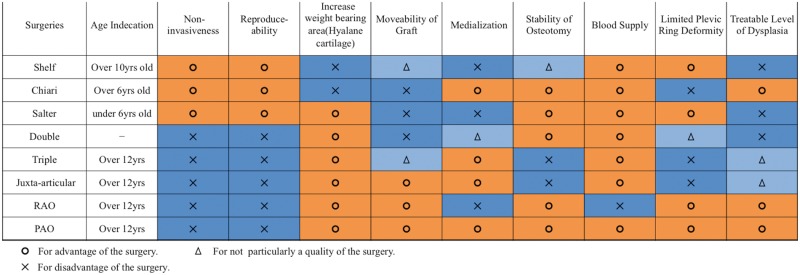

Clohisy *et al*. [88] have indicated the indications for the PAO for good results as to be adolescent or adult patients with a dysplastic hip, with a congruent hip with abduction, adequate range of motion (105° flexion, 30° abduction) and minimal or no secondary osteoarthritic change.

#### Assessing the Bernese PAO

It is an invasive surgery. Weight bearing area of Hyaline cartilage is increased. The movability of the graft is good. Medialization of the join is a feature of this osteotomy. The osteotomy is stable. The blood supply to the graft is good. Deformity of the pelvic ring is limited. The level of dysplasia that can be treated is broad, but does have its limitations (Table VIII).

## DISCUSSION

In this manuscript, the authors’ assessed the relative abilities of the various described procedures in achieving ideal goals and characteristics of a procedure for the treatment of hip dysplasia, and have summarized the results in Table VIII. It can be observed that at the expense of some increase in invasiveness, the PAO has evolved to achieve many of these theoretical ideals, becoming the current gold standard in the treatment of the dysplastic hip in the skeletally mature patient. Even so, the Shelf operation has its advantage as not being as technically demanding and invasive a surgery and still can be considered a surgical option in cases with minor dislocation and limited osteoarthritic change. As described, the broad indications for the Chiari osteotomy may make it a very important option in the difficult, neglected congenital dislocation or the hip with secondary osteoarthritis where the other pelvic osteotomies are not indicated, still considerations toward the drawbacks of the surgery must be made. Thus, essentially, the role for the Chiari osteotomy appears to be a salvage procedure, and with current, more modern anatomic techniques, the Chiari has been replaced for straightforward, primary management of the dysplastic hip. The RAO theoretically seems to have some disadvantages but the many superb long-term results are proof that in the hands of the experienced surgeon this surgery is a powerful tool for the treatment of the dysplastic hip.

**Fig. 3.**
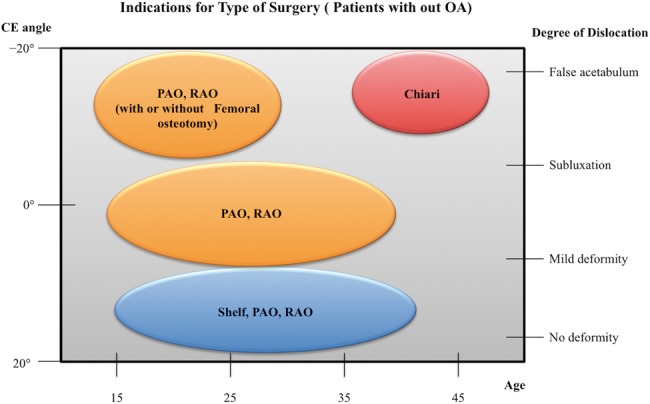
Indications for type of surgery.

This article describes the evidence-based indications for the different osteotomies described for DDH in Fig. 3. The young patient with little to no osteoarthritis, minor dysplasia and no subluxation is a good candidate for the PAO, RAO and the Shelf operation. The older patient with an inferior CE angle, subluxation, incongruency with or without osteoarthritis would be a candidate for the Chiari osteotomy. All other patients would be considered candidates for a PAO or a RAO. In some cases, when it is difficult to obtain joint congruency, a femoral osteotomy in combination with a PAO or a RAO is a consideration but this scenario is beyond the scope of this review. It is difficult to generalize the indication for all the different scenarios as the age and level of osteoarthritis also have a great impact on the results of the surgery. The age, level of dysplasia, level of subluxation, the congruity and level of osteoarthritis in each patient as well as the experience of the surgeon are important factors that must be thoroughly assessed when determining the treatment of choice.

As this was a review of the long-term results of each surgery, we were not able to assess the differences between surgeries in their immediate postoperation rehabilitation phases, where in modern day treatment minimal invasiveness and short-term rehabilitation has become an emphasis. In the modern era the demands and standers of the patient and the surgeon have drastically increased, as improvement in not only pain but also restoration of an active physical life is desired. The THA prosthesis and surgical techniques have also improved making the emphasis on the longevity of the initial joint preservation surgery not as important a factor as it may have been in the past. We understand these changes in the meaning of what a successful surgery is make it difficult to compare the results of past surgeries to present standards. Even though the endpoint of the surgery may differ, assessing their long-term results still gives us an assumption as to how long the patients were relatively satisfied with their surgery. We believe it is meaningful to assess the many surgeries of our predecessors and see how their results have done over a prolong period of time and by doing so hopefully open the possibility to new techniques or ideas for improvements in the future.

This review has focused on the long-term results of augmental osteoplasties and pelvic osteotomies, other than the PAO, that have been used to treat the dysplastic hip in the skeletally mature patient. From its characteristics, the Bernese PAO would be the surgery of choice for this entity. However, many surgeons have shown other surgeries such as the Shelf operation, RAO and Chiari osteotomy have proven over time that when the indications are correctly met and surgery is conducted properly excellent to good results can be expected even in the long-term, and can still be considered to have a place in the treatment of hip dysplasia.

## CONFLICT OF INTEREST STATEMENT

None declared.

## References

[1] Ganz R, Klaue K, Vinh TS A new periacetabular osteotomy for the treatment of hip dysplasias. Technique and preliminary results. Clin Orthop Relat Res 1988; 232: 26–36. 3383491

[2] Salter RB The classic innominate osteotomy in the treatment of congenital dislocation and subluxation of the hip. J Bone Joint Surg Br 1961; 43-B: 518–30.

[3] Laurent LE Congenital dislocation of the hip: acetabular inclination and femoral torsion; primary results of closed reduction checked by arthrography and of open treatment with transposition of the lieopsoas muscle. Acta Chir Scand Suppl 1953; 179: 1–133. 13113807

[4] Langenskiold F On the transposition of the ileopsoas-muscle in operative reduction of congenital hip-dislocation. Acta Orthop Scand 1953; 22: 295–9. 13091855

[5] Böhm P, Brzuske A Salter innominate osteotomy for the treatment of developmental dysplasia of the hip in children: results of seventy-three consecutive osteotomies after twenty-six to thirty-five years of follow-up. J Bone Joint Surg Am 2002; 84-A: 178–86. 11861722

[6] Thomas SR, Wedge JH, Salter RB Outcome at forty-five years after open reduction and innominate osteotomy for late-presenting developmental dislocation of the hip. J Bone Joint Surg Am 2007; 89: 2341–50. 17974875 10.2106/JBJS.F.00857

[7] Deng Wiktor Osteotomia trans-iliakalna w leczeniu wrodzonej dysplazji biodra. Chir Narz Ruchu Ortop Pol 1974; 39: 601–13. 4422569

[8] Pemberton PA Pericapsular congenital osteotomy subluxation of the ilium dislocation for treatment of the of hip. J Bone Joint Surg Am 1965; 47-A: 65–86. 14256975

[9] Salter RB, Feild P The effects of continuous compression on living articular cartilage. An experimental invextigation. J Bone Joint Surg Am 1960; 42-A, 31.

[10] Root L, Laplaza FJ, Brourman SN The severely unstable hip in cerebral palsy. Treatment with open reduction, pelvic osteotomy, and femoral osteotomy with shortening. J Bone Joint Surg Am 1995; 77: 703–12. 7744895 10.2106/00004623-199505000-00006

[11] Eyre-Brook A, Jones D, Harris F Pemberton’s acetabuloplasty for congenital dislocation or subluxation of the hip. J Bone Joint Surg Br 1978; 60-B: 18–24. 10.1302/0301-620X.60B1.627574 627574

[12] Faciszewski T, Kiefer GN, Coleman SS Pemberton osteotomy for residual acetabular dysplasia in children who have congenital dislocation of the hip. J Bone Joint Surg Am 1993; 75: 643–9. 8501078 10.2106/00004623-199305000-00002

[13] Sutherland D, Greenfield R Double innominate osetotomy. J Bone Joint Surg Am 1977; 59-A: 1082–91. 591540

[14] Sutherland D, Moore M Clinical and radiographic outcome of patients treated with double innominate osteotomy for congenital hip dysplasia. J Pediatr Orthop 1991; 11: 143–8. 2010510 10.1097/01241398-199103000-00001

[15] Le Coeur P Corrections des défauts d’orientation de l’articulation coxofé- morale par ostéotomie de l’os iliaque. Rev Chir Orthop 1965; 51: 211–2.

[16] Hopf A Hip acetabular displacement by double pelvic osteotomy in the treat- ment of hip joint dysplasia and sublux- ation in young people and adults. Z Orthop Ihre Grenzgeb 1966; 101: 559–86. 4230758

[17] Steel H Triple osteotomy of the innominate bone. J Bone Joint Surg Am 1973; 55-A: 343–50. 4572223

[18] Sanchez-sotelo J, Trousdale RT, Berry DJ Surgical Treatment of developmental dysplasia of the hip in adults: I. Nonarthroplasty options. J Am Acad Orthop Surg 2002; 10:321–33. 12374483 10.5435/00124635-200209000-00004

[19] Tönnis D, Behrens K, Tscharani F A modified technique of the triple pelvic osteotomy: early results. J Pediatr Orthop 1981; 1: 241–9. 7334101 10.1097/01241398-198111000-00001

[20] Carlioz H, Khouri N, Hulin P Ostéotomie pelvienne triple juxta-cotyloïdienne. Rev Chir Orthop 1982; 68: 497–501. 6220445

[21] Guille JT, Forlin E, Jumar SJ Triple osteotomy of the innominate bone in treatment of developmental dysplasia of the hip. J Pediatr Orthop 1992; 12: 718–21. 1452738 10.1097/01241398-199211000-00003

[22] Dungl P, Rejholec M, Chomiak J The role of triple pelvic osteotomy in therapy of residual hip dysplasia and sequel of AVN: long-term experience. Hip Int 2007; 17(Suppl. 5): S51–64. 19197885

[23] Janssen D, Kalchschmidt K, Katthagen B-D Triple pelvic osteotomy as treatment for osteoarthritis secondary to developmental dysplasia of the hip. Int Orthop 2009; 33: 1555–9. 19214509 10.1007/s00264-008-0718-5 PMC2899173

[24] Van Stralen RA, van Hellemondt GG, Ramrattan NN Can a triple pelvic osteotomy for adult symptomatic hip dysplasia provide relief of symptoms for 25 years? *Clin**. *Orthop Relat Res 2013; 471: 584–90. 10.1007/s11999-012-2701-0 PMC3549168 23179122

[25] Wagner H Osteotomies for congenital hip dislocation. hip. Proc Fourth Open Sci Meet Hip Soc St Louis 1976; 10: 46–66.

[26] Eppright R Dial osteotomy of the acetabulum in the treatment of dysplasia of the hip. J Bone Joint Surg 1975; 57: 1170–4.

[27] Ninomiya S, Tagawa H Rotational acetabular osteotomy for the dysplastic hip. J Bone Joint Surg Am 1984; 66: 430–6. 6699061

[28] Trousdale RT, Ekkernkamp A, Ganz R Periacetabular and intertrochanteric osteotomy for the treatment of osteoarthrosis in dysplastic hips. J Bone Joint Surg Am 1995; 77: 73–85. 7822358 10.2106/00004623-199501000-00010

[29] Millis MB, Murphy SB, Poss R Display settings: osteotomies about the hip for the prevention and treatment of publication types, MeSH terms PubMed commons PubMed commons home how to join PubMed commons. Instr Course Lect 1996; 45: 209–26. 8727740

[30] Matsui M, Masuhara K, Nakata K Early deterioration after modified rotational acetabular osteotomy for the dysplastic hip. J Bone Joint Surg Br 1997; 79-B: 220–4. 10.1302/0301-620x.79b2.7202 9119846

[31] Murphy SB, Millis MB, Hall JE Surgical correction of acetabular dysplasia in the adult. Clin Orthop Relat Res 1999; 363: 38–44. 10379303

[32] Nozawa M, Shitoto K, Matsuda K Rotational acetabular osteotomy for acetabular dysplasia. J Bone Joint Surg Br 2002; 84-B: 59–65. 10.1302/0301-620x.84b1.12299 11837834

[33] Schramm M, Hohmann D, Radespiel-Troger M Treatment of the dysplastic acetabulum with Wagner spherical osteotomy. A study of patients followed for a minimum of twenty years. J Bone Joint Surg Am 2003; 85-A: 808–14. 12728029 10.2106/00004623-200305000-00006

[34] Schramm M, Hohmann D, Pitto RP The Wagner spherical osteotomy of the acetabulum. J Bone Joint Surg Am 2004; 86-A(Suppl. 1): 73–80. 10.2106/00004623-200403001-00010 14996924

[35] Miller NH, Krishnan SG, Kamaric E Long-term results of the dial osteotomy in the treatment of high-grade acetabular dysplasia. Clin Orthop Relat Res 2005; 433: 115–23. 10.1097/01.blo.0000153992.17554.67 15805946

[36] Nakamura S, Ninomiya S Long-term outcome of rotational acetabular osteotomy: 145 hips followed for PubMed commons. Acta Orthop Scand 1998; 69: 259–65. 9703399 10.3109/17453679809000926

[37] Takatori Y, Ninomiya S, Nakamura S Long-term results of rotational acetabular osteotomy in patients with slight narrowing of the joint space on preoperative radiographic findings. J Orthop Sci 2001; 6: 137–40. 11484099 10.1007/s007760100061

[38] Hasegawa Y, Iwase T, Kitamura S Eccentric rotational acetabular osteotomy for acetabular dysplasia and osteoarthritis. J Bone Joint Surg. Am 2014; 96-A: 1975–82. 10.2106/JBJS.M.01563 25471912

[39] Okano K, Enomoto H, Osaki M Rotational acetabular osteotomy for advanced osteoarthritis secondary to developmental dysplasia of the hip. J Bone Joint Surg Br 2008; 90: 23–6. 18160494 10.1302/0301-620X.90B1.19665

[40] Yasunaga Y, Yamasaki T, Ochi M Patient selection criteria for periacetabular osteotomy or rotational acetabular osteotomy. Clin Orthop Relat Res 2012; 470: 3342–54. 22895690 10.1007/s11999-012-2516-z PMC3492602

[41] Yamaguchi J, Hasegawa Y, Kanoh T Similar survival of eccentric rotational acetabular osteotomy in patients younger and older than 50 years. Clin Orthop Relat Res 2009; 467: 2630–7. 19424675 10.1007/s11999-009-0866-y PMC2745461

[42] Yasunaga Y, Ochi M, Shimogaki K Rotational acetbular osteotomy for hip dysplasia. Acta Orthop Scand 2004; 75: 10–5. 15022799 10.1080/00016470410001708020

[43] Okano K, Enomoto H, Osaki M Outcome of rotational acetabular osteotomy for early hip osteoarthritis secondary to dysplasia related to femoral head shape. Acta Orthop 2008; 79: 12–7. 18283566 10.1080/17453670710014699

[44] Ito H, Tanino H, Yamanaka Y Intermediate to long-term results of periacetabular osteotomy in patients younger and older than forty years of age. J Bone Joint Surg Am 2011; 93: 1347–54. 21792502 10.2106/JBJS.J.01059

[45] Yasunaga Y, Takahashi K, Ochi M Rotational acetabular osteotomy in patients forty-six years of age or older: comparison with younger patients. J Bone Joint Surg Am 2003; 85-A: 266–72. 12571304 10.2106/00004623-200302000-00013

[46] Nozawa M, Maezawa K, Matsuda K Rotational acetabular osteotomy for advanced osteoarthritis of the hip joint with acetabular dysplasia. Int Orthop 2009; 33: 1549–53. 18853158 10.1007/s00264-008-0657-1 PMC2899188

[47] Köng F Osteoplastische Behandlung der kongenital Huftgelenkuxation. Verh Deutsch Ges Chir 1891; 20: 75–80.

[48] Albee FH The Bone graft wedge: its use in the treatment of relapsing acquired and congenital dislocation of the hip. NY Med J 1915; 102: 433–5.

[49] Lance P Herstellung eines osteoplastischen Pfannen daches bei angeborenen Verrenkung und Subluxationen der Hufte. Press Med 1925: 945.

[50] Howorth MB Congenital dislocation of the hip. Ann Surg 1947; 125: 216–36. 17858926 10.1097/00000658-194702000-00008 PMC1803160

[51] Crego CH, Schwartzmann JR Follow-up study of the early treatment of congenital dislocation of the hip. J Bone Joint Surg Am 1948; 30A: 428–42. 18912305

[52] Gill AB The end results of early treatment of congenital dislocation of the hip, with an inquiry into the factors that determine the results. J Bone Joint Surg Am 1948; 30A: 442–53. 18912306

[53] Wiberg G Shelf operation in congenital dysplasia of the acetabulum and in subluxation and dislocation of the hip. J Bone Joint Surg Am 1953; 35-A: 65–80. 13022708

[54] Spitzy H Kunstliche Pfannendachbildung. Zeitschr f Orthop 1923; 43: 284–94.

[55] Ghormley RK Use of the anterior superior spine and crest of ilium in surgery of the hip. J Bone Joint Surg 1931; 13: 784–98.

[56] Bosworth DM, Fielding JW, Liebler WA Hip shelves in children. J Bone Joint Surg 1960; 42: 1223–38.

[57] Bosworth DM, Fielding JW, Ishizuka T Hip-shelf operation in adults. J Bone Joint Surg 1961; 43: 93–106.

[58] Heyman CH Long-term resluts following a bone-shelf operation for congenial and other dislocations of the hip in children. J Bone Joint Surg Am 1963; 45: 1113–46. 14077978

[59] Wilson JC Surgical treatment of the dysplastic acetabulum in adolescence. Clin Orthop Relat Res 1974; 98: 137–45. 10.1097/00003086-197401000-00015 4817223

[60] Wainwright D The shelf operation for hip dysplasia in adolescence. J Bone Joint Surg Br 1976; 58: 159–63. 932076 10.1302/0301-620X.58B2.932076

[61] Chiari K Medial displacement osteotomy of the pelvis. Clin Orthop Relat Res 1974; 98: 55–71. 10.1097/00003086-197401000-00008 4817245

[62] Hiranuma S, Higuchi F, Inoue A Changes in the interposed capsule after Chiari osteotomy. An experimental study on rabbits with acetabular dysplasia. J Bone Joint Surg Br 1992; 74: 463–7. 1587903 10.1302/0301-620X.74B3.1587903

[63] Hirose S, Otsuka H, Morishima T Long-term outcomes of shelf acetabuloplasty for developmental dysplasia of the hip in adults: a minimum 20-year follow-up study. J Orthop Sci 2011; 16: 698–703. 21915667 10.1007/s00776-011-0159-7 PMC3230761

[64] Staheli LT, Chew DE Slotted acetabular augmentation in childhood and adolescence. J Pediatr Orthop 1992; 12: 569–80. 1517414

[65] White RE, Sherman FC The hip-shelf procedure. J Bone Joint Surg Am 1980; 62-A: 928–32. 7430180

[66] Fawzy E, Mandellos G, De Steiger R Is there a place for shelf acetabuloplasty in the management of adult acetabular dysplasia? A survivorship study. J Bone Joint Surg Br 2005; 87: 1197–202. 16129741 10.1302/0301-620X.87B9.15884

[67] Migaud H, Chantelot C, Giraud F Long-term survivorship of hip shelf arthroplasty and Chiari osteotomy in adults. Clin Orthop Relat Res 2004; 418: 81–6. 10.1097/00003086-200401000-00014 15043097

[68] Bickel WH, Brevis JS Shelf operation for congenital subluxation and dislocation of the hip. Clin Orthop Relat Res 1975; 106: 27–34. 10.1097/00003086-197501000-00004 1126084

[69] Love BR, Stevens PM, Williams PF A long-term review of shelf arthroplasty. J Bone Joint Surg Br 1980; 62-B: 321–5. 10.1302/0301-620X.62B3.7410463 7410463

[70] Saito S, Takaoka K, Ono K Tectoplasty for painful dislocation or subluxation of the hip. J Bone Joint Surg Am 1986; 68-B: 55–60. 10.1302/0301-620X.68B1.3510216 3510216

[71] Summers BN, Turner A, Wynn-Jones C The shelf operation in the management of late presentation of congenital bip dysplasia. J. Bone Joint Surg Am 1988; 70-B: 63–8. 10.1302/0301-620X.70B1.3276702 3276702

[72] Bartoníček J, Vávra J, Chochola A Bosworth hip shelf arthroplasty in adult dysplastic hips: ten to twenty three year results. Int Orthop 2012; 36: 2425–31. 23093289 10.1007/s00264-012-1665-8 PMC3508042

[73] Nishimatsu H, Iida H, Kawanabe K The modified Spitzy shelf operation for patients with dysplasia of the hip. J Bone Joint Surg Br 2002; 84-B: 647–52. 10.1302/0301-620x.84b5.12732 12188478

[74] Chiari K Medial displacement osteotomy of the pelvis. Clin Orthop Relat Res 1974; 98: 146–50. 10.1097/00003086-197401000-00008 4817245

[75] Reynolds D Chiari innominate osteotomy in adluts techniques, indications and contra-indications. J Bone Joint Surg Br 1986; 68-B: 45–54. 10.1302/0301-620X.68B1.3941141 3941141

[76] Calvert P, August A, Albert J The Chiari pelvic oseteotomy. J Bone Joint Surg Br 1987; 69-B: 551–5. 10.1302/0301-620X.69B4.3611157 3611157

[77] Windhager R, Pongracz N, Schonecher W Chiari osteotomy for congenital dislocation and subluxation of the hip, Ruslts after 20 to 34 years follow-up. J Bone Joint Surg Br 1991; 73-B: 890–5. 10.1302/0301-620X.73B6.1955430 1955430

[78] Lack W, Windhager R, Kutschera H Chiari plevic osteotomy for osteoarthritis secondary to hip dysplasia, Indications and long-term results. J Bone Joint Surg Br 1991; 73-B: 229–34. 10.1302/0301-620X.73B2.2005145 2005145

[79] Ohashi H, Hirohashi K, Yamano Y Factors influencing the outcome of Chiari pelvic osteotomy: a long-term follow-up. J Bone Joint Surg Br 2000; 82: 517–25. 10855874 10.1302/0301-620x.82b4.9583

[80] Yanagimoto S, Hotta H, Izumida R Long-term results of Chiari pelvic osteotomy in patients with developmental dysplasia of the hip: indications for Chiari pelvic osteotomy according to disease stage and femoral head shape. J Orthop Sci. 2005; 10: 557–63. 16307180 10.1007/s00776-005-0942-4

[81] Kotz R, Chiari C, Hofstaetter JG Long-term experience with Chiari’s osteotomy. Clin Orthop Relat Res 2009; 467: 2215–20. 19521741 10.1007/s11999-009-0910-y PMC2866931

[82] Kim K-I, Cho Y-J, Ramteke AA Peri-acetabular rotational osteotomy with concomitant hip arthroscopy for treatment of hip dysplasia. J Bone Joint Surg Br 2011; 93: 732–7. 21586769 10.1302/0301-620X.93B6.25809

[83] Ross JR, Zaltz I, Nepple JJ Arthroscopic disease classification and interventions as an adjunct in the treatment of acetabular dysplasia. Am. J Sports Med 2011; 39(Suppl): 72S–8S. 21709035 10.1177/0363546511412320

[84] Siebenrock KA, Scholl E, Lottenbarch M Bernese periacetbular osetotmy. Clin Orthop Relat Res 1999; 363: 9–20. 10379300

[85] Steppacher SD, Tannast M, Ganz R Mean 20-year followup of Bernese periacetabular osteotomy. Clin Orthop Relat Res 2008; 466: 1633–44. 18449617 10.1007/s11999-008-0242-3 PMC2505253

[86] Albers CE, Steppacher SD, Ganz R Impingement adversely affects 10-year survivorship after periacetabular osteotomy for DDH. Clin Orthop Relat Res 2013; 471: 1602–14. 23354462 10.1007/s11999-013-2799-8 PMC3613512

[87] Kralj M, Mavcic B, Antolic V The Bernese periacetabular osteotomy: clinical, radiographic and mechanical 7-15-year follow-up of 26 hips. Acta Orthop 2005; 76: 833–40. 16470438 10.1080/17453670510045453

[88] Clohisy BJC, Barrett SE, Gordon JE Periacetabular osteotomy in the treatment of severe acetabular dysplasia surgical technique. J Bone Joint Surg Am 2006; 88-A: 65–83. 10.2106/JBJS.E.00887 16510801

